# Insuficiência Cardíaca no Brasil: Como Podemos Melhorar sua História Natural?

**DOI:** 10.36660/abc.20240644

**Published:** 2024-11-07

**Authors:** Filipe Ferrari, Arthur Proença Rossi, Igor Rafael Miranda Ferreira Santander

**Affiliations:** 1 Universidade Federal do Rio Grande do Sul Porto Alegre RS Brasil Programa de Pós-Graduação em Cardiologia e Ciências Cardiovasculares – Universidade Federal do Rio Grande do Sul, Hospital de Clínicas de Porto Alegre, Porto Alegre, RS – Brasil; 2 Universidade Federal do Rio Grande do Sul Hospital de Clínicas de Porto Alegre Porto Alegre RS Brasil Universidade Federal do Rio Grande do Sul, Hospital de Clínicas de Porto Alegre, Porto Alegre, RS – Brasil; 3 Hospital Nova Esperança João Pessoa PB Brasil Programa de Residência em Clínica Médica, Hospital Nova Esperança, João Pessoa, PB – Brasil

**Keywords:** Insuficiência Cardíaca, Insuficiência Cardíaca Sistólica, Brasil

A insuficiência cardíaca (IC) é uma síndrome complexa, com altas taxas de morbidade e mortalidade em todo o mundo.^1,2^ Apesar dos esforços significativos, incluindo vários ensaios clínicos recentes envolvendo dezenas de milhares de pacientes e diversas terapias farmacológicas,^3-9^ reduzir a carga da IC continua sendo um desafio.

Em 2021, dados epidemiológicos estimaram que aproximadamente 57 milhões de pessoas foram afetadas por IC globalmente,^10^ e sua prevalência está projetada para aumentar em 34% nas próximas décadas.^11^ Pacientes hospitalizados devido ao agravamento da IC estão entre os grupos de maior risco, enfrentando taxas elevadas de readmissão e mortalidade, particularmente durante o período vulnerável logo após a alta.^12^ O fardo econômico da IC também é significativo; até 2030, os custos relacionados à IC nos EUA estão projetados para exceder US$ 70 bilhões anualmente.^13^

Em resposta às percepções em evolução, a Sociedade Europeia de Cardiologia introduziu uma nova classificação para pacientes com fração de ejeção (FE) do ventrículo esquerdo entre 40% e 49%, designando-os como portadores de IC com FE levemente reduzida (ICFEIr), substituindo o termo anterior "IC com FE intermediária". A IC com FE reduzida (ICFEr) continua a ser classificada como FE <40%, enquanto a IC com fração de ejeção preservada (ICFEp) é definida como FE >50%.^14^ Estima-se que a ICFEIr seja responsável por até 25% de todos os casos de IC.^15^

A epidemiologia da IC é bem documentada na Europa e nos Estados Unidos; no entanto, sua prevalência e fatores prognósticos são menos compreendidos em outras partes do mundo. No Brasil, um vasto país com significativa diversidade étnica e disparidades sociais, há dados limitados sobre potenciais diferenças regionais no prognóstico e tratamento da IC. Essa lacuna dificulta o desenvolvimento de estratégias de gerenciamento mais eficazes e específicas para cada região, adaptadas à população brasileira.

Um registro multinacional recente de IC, com mais de 23.000 participantes de 40 países em cinco continentes, acompanhados por uma mediana de 2 anos, descobriu que as taxas de mortalidade padronizadas por idade e sexo eram menores em países de alta renda.^16^ A taxa de letalidade de casos em 30 dias após a primeira hospitalização seguiu uma tendência semelhante: 7% em países de alta renda, 10% em países de renda média-alta, 21% em países de renda média-baixa e 32% em países de baixa renda. Apoiando essas descobertas, as evidências mostram que as taxas de mortalidade de um ano em pacientes com IC permanecem altas em países de baixa e média renda, atingindo 34% na África, 23% na Índia, 15% no Sudeste Asiático, 9% na América do Sul e 7% na China.^17^ Esses dados sugerem que os países de baixa renda apresentam piores resultados em IC, provavelmente devido ao acesso limitado a cuidados de saúde de qualidade, diferenças nas características dos pacientes e uso reduzido de terapias de IC modificadoras da doença. De fato, o tratamento combinado com medicamentos comprovadamente redutores da morbidade e mortalidade pode alterar significativamente a história natural da doença.^18^ Antes da década de 1990, considerada o início da era moderna do tratamento da IC, entre 60% e 70% dos pacientes morriam dentro de cinco anos após o diagnóstico.^19^

Pesquisas sobre IC também foram conduzidas no Brasil. Por exemplo, Arruda et al.^20^ relataram dados importantes sobre mortalidade por IC entre 1998 e 2019, identificando áreas geográficas com as maiores taxas de mortalidade. Aproximadamente 568.000 mortes foram registradas entre adultos com mais de 50 anos de idade, resultando em uma taxa média de mortalidade de 75,5 por 100.000 habitantes. É importante ressaltar que a mortalidade por IC mostrou uma tendência crescente na região norte, ressaltando a necessidade de intervenções direcionadas em áreas com resultados mais precários. Da mesma forma, o estudo BREATHE,^21^ que incluiu mais de 1.200 pacientes hospitalizados com IC em 51 hospitais públicos e privados em 21 cidades brasileiras, forneceu informações valiosas sobre IC no país. No entanto, esses estudos não ofereceram informações detalhadas sobre características clínicas, tratamentos farmacológicos, disponibilidade financeira ou programas de financiamento governamental para acesso à terapia medicamentosa otimizada em diferentes regiões do Brasil.

O desafio continua sendo identificar quais regiões estão com baixo desempenho no tratamento da IC ou onde os pacientes apresentam maior risco de progressão para IC avançada. Nesta edição do ABC, Freitas et al.^22^ apresentaram um protocolo para um estudo de coorte multicêntrico prospectivo com o objetivo de avaliar dados de 2.500 pacientes com ICFEr ou ICFEIr em 30 centros em 23 unidades federativas brasileiras. O desenho prospectivo deste estudo e a coleta abrangente de dados – incluindo histórico médico prévio (por exemplo, comorbidades, tabagismo, histórico de doenças tropicais negligenciadas), sinais e sintomas (por exemplo, classe funcional NYHA, edema periférico, ascite, hepatomegalia), achados ecocardiográficos (por exemplo, doenças valvares, válvula protética), resultados de exames laboratoriais (por exemplo, NT-proBNP, eletrólitos, lipídios) e adesão ao tratamento médico orientado por diretrizes de IC (por exemplo, betabloqueadores, inibidores do sistema renina-angiotensina-aldosterona, inibidores de SGLT2) – aumentam significativamente seu impacto potencial.

Esta pesquisa representa um passo crítico para a ciência brasileira na descoberta de variações regionais e socioeconômicas no prognóstico da IC. Em última análise, visa desenvolver estratégias mais eficazes para combater essa condição devastadora. Além disso, pode ajudar a identificar populações de alto risco e orientar intervenções personalizadas e direcionadas.

Em resumo, este estudo pode contribuir com dados valiosos para o crescente corpo de literatura sobre IC. Além de expandir nossa compreensão sobre IC no Brasil, ele terá implicações práticas para melhorar a saúde pública e o atendimento clínico. Embora ainda haja muito trabalho a ser feito, essas descobertas podem ajudar a informar melhores estratégias para reduzir a morbidade e a mortalidade por IC no Brasil. A hora de agir é agora.
