# Supplementary material for: Insuficiência Cardíaca no Brasil: Como Podemos Melhorar sua História Natural?
Source: Arq Bras Cardiol. 2024 Nov 7;121(10):e20240644. [Article in Portuguese] doi: 10.36660/abc.20240644 (PMC11634228; doi:10.36660/abc.20240644)
Supplement: Translation

# Translation: Heart Failure in Brazil: How Can We Improve its Natural History?

**DOI:** 10.36660/abc.20240644i

**Authors:** Filipe Ferrari, Arthur Proença Rossi, Igor Rafael Miranda Ferreira Santander

**Affiliations:** 1 Porto Alegre RS Brazil Programa de Pós-Graduação em Cardiologia e Ciências Cardiovasculares – Universidade Federal do Rio Grande do Sul, Hospital de Clínicas de Porto Alegre, Porto Alegre, RS – Brazil; 2 Porto Alegre RS Brazil Universidade Federal do Rio Grande do Sul, Hospital de Clínicas de Porto Alegre, Porto Alegre, RS – Brazil; 3 João Pessoa PB Brazil Programa de Residência em Clínica Médica, Hospital Nova Esperança, João Pessoa, PB – Brazil

**Keywords:** Heart Failure, Systolic Heart Failure, Brazil

Heart failure (HF) is a complex syndrome with high morbidity and mortality rates worldwide.^1,2^ Despite significant efforts, including several recent clinical trials involving tens of thousands of patients and various pharmacologic therapies,^3-9^ reducing the burden of HF remains a challenge.

As of 2021, epidemiological data estimated that approximately 57 million people were affected by HF globally,^10^ and its prevalence is projected to rise by 34% in the coming decades.^11^ Patients hospitalized due to worsening HF are among the highest-risk groups, facing elevated rates of readmission and mortality, particularly during the vulnerable period shortly after discharge.^12^ The economic burden of HF is also significant; by 2030, HF-related costs in the U.S. are projected to exceed $70 billion annually.^13^

In response to evolving insights, the European Society of Cardiology introduced a new classification for patients with a left ventricular ejection fraction (EF) between 40% and 49%, designating them as having HF with mildly reduced EF (HFmrEF), replacing the previous term "HF with mid-range EF". HF with reduced EF (HFrEF) continues to be classified as EF <40%, while HF with preserved ejection fraction (HFpEF) is defined as EF >50%.^14^ It is estimated that HFmrEF accounts for up to 25% of all HF cases.^15^

The epidemiology of HF is well-documented in Europe and the United States; however, its prevalence and prognostic factors are less understood in other parts of the world. In Brazil, a vast country with significant ethnic diversity and social disparities, there is limited data on potential regional differences in HF prognosis and treatment. This gap hinders the development of more effective, region-specific management strategies tailored to the Brazilian population.

A recent multinational HF registry with over 23,000 participants from 40 countries across five continents, followed for a median of 2 years, found that age- and sex-standardized mortality rates were lower in high-income countries.^16^ The 30-day case fatality rate after the first hospitalization followed a similar trend: 7% in high-income countries, 10% in upper-middle-income countries, 21% in lower-middle-income countries, and 32% in low-income countries. Supporting these findings, evidence shows that one-year mortality rates in HF patients remain high in low- and middle-income countries, reaching 34% in Africa, 23% in India, 15% in Southeast Asia, 9% in South America, and 7% in China.^17^ These data suggest that lower-income countries experience worse HF outcomes, likely due to limited access to quality healthcare, differences in patient characteristics, and reduced use of disease-modifying HF therapies. In fact, combined treatment with medications proven to reduce morbidity and mortality can significantly alter the natural history of the disease.^18^ Before the 1990s, considered the beginning of the modern era of HF treatment, between 60% and 70% of patients died within five years of diagnosis.^19^

Research on HF has also been conducted in Brazil. For example, Arruda et al.^20^ reported important data on HF mortality between 1998 and 2019, identifying geographic areas with the highest mortality rates. Approximately 568,000 deaths were recorded among adults over 50 years of age, resulting in an average mortality rate of 75.5 per 100,000 inhabitants. Importantly, HF mortality showed an increasing trend in the northern region, underscoring the need for targeted interventions in areas with poorer outcomes. Similarly, the BREATHE study,^21^ which included over 1,200 patients hospitalized with HF across 51 public and private hospitals in 21 Brazilian cities, provided valuable insights into HF in the country. However, these studies did not offer detailed information on clinical characteristics, pharmacological treatments, financial availability, or government funding programs for access to optimized drug therapy across different regions of Brazil.

The challenge remains in identifying which regions are underperforming in managing HF or where patients are at higher risk of progressing to advanced HF. In this issue of the *ABC*, Freitas et al.^22^ presented a protocol for a prospective multicenter cohort study aimed at evaluating data from 2,500 patients with HFrEF or HFmrEF across 30 centers in 23 Brazilian federative units. This study's prospective design and comprehensive data collection – including prior medical history (e.g., comorbidities, smoking, history of neglected tropical diseases), signs and symptoms (e.g., NYHA functional class, peripheral edema, ascites, hepatomegaly), echocardiographic findings (e.g., valvular diseases, prosthetic valve), laboratory test results (e.g., NT-proBNP, electrolytes, lipids), and adherence to HF guideline-directed medical treatment (e.g., beta-blockers, renin–angiotensin aldosterone system inhibitors, SGLT2 inhibitors) – significantly enhance its potential impact.

This research represents a critical step forward for Brazilian science in uncovering regional and socioeconomic variations in HF prognosis. Ultimately, it aims to develop more effective strategies to combat this devastating condition. Additionally, it may help identify high-risk populations and guide personalized, targeted interventions.

In summary, this study could contribute valuable data to the growing body of HF literature. Beyond expanding our understanding of HF in Brazil, it will have practical implications for improving public health and clinical care. While much work remains, these findings could help inform better strategies to reduce HF morbidity and mortality in Brazil. The time to act is now.
